# Functional and Immunological Relevance of *Anaplasma marginale* Major Surface Protein 1a Sequence and Structural Analysis

**DOI:** 10.1371/journal.pone.0065243

**Published:** 2013-06-11

**Authors:** Alejandro Cabezas-Cruz, Lygia M. F. Passos, Katarzyna Lis, Rachel Kenneil, James J. Valdés, Joana Ferrolho, Miray Tonk, Anna E. Pohl, Libor Grubhoffer, Erich Zweygarth, Varda Shkap, Mucio F. B. Ribeiro, Agustín Estrada-Peña, Katherine M. Kocan, José de la Fuente

**Affiliations:** 1 University of South Bohemia, Faculty of Science and Biology Centre of the Academy of Sciences of the Czech Republic, Parasitology Institute, České Budějovice, Czech Republic; 2 Institute for Comparative Tropical Medicine and Parasitology, Ludwig-Maximilians-Universität, München, Germany; 3 Departamento de Medicina Veterinária Preventiva, Escola de Veterinária, Universidade Federal de Minas Gerais, Belo Horizonte, Minas Gerais, Brazil; 4 Pirbright Laboratory, The Pirbright Institute, Pirbright, Surrey, United Kingdom; 5 Division of Parasitology, Kimron Veterinary Institute, Bet Dagan, Israel; 6 Departamento de Parasitologia, Universidade Federal de Minas Gerais, Belo Horizonte, Minas Gerais, Brazil; 7 Facultad de Veterinaria, Universidad de Zaragoza, Zaragoza, Spain; 8 Department of Veterinary Pathobiology, Center for Veterinary Health Sciences, Oklahoma State University, Stillwatert, Oklahoma, United States of America; 9 Sanidad y Biotecnología, Instituto de Investigación de Recursos Cinegéticos, IREC-CSIC-UCLM-JCCM, Ciudad Real, Spain; Kansas State University, United States of America

## Abstract

Bovine anaplasmosis is caused by cattle infection with the tick-borne bacterium, *Anaplasma marginale*. The major surface protein 1a (MSP1a) has been used as a genetic marker for identifying *A. marginale* strains based on N-terminal tandem repeats and a 5′-UTR microsatellite located in the msp1a gene. The MSP1a tandem repeats contain immune relevant elements and functional domains that bind to bovine erythrocytes and tick cells, thus providing information about the evolution of host-pathogen and vector-pathogen interactions. Here we propose one nomenclature for A. marginale strain classification based on MSP1a. All tandem repeats among *A. marginale* strains were classified and the amino acid variability/frequency in each position was determined. The sequence variation at immunodominant B cell epitopes was determined and the secondary (2D) structure of the tandem repeats was modeled. A total of 224 different strains of *A. marginale* were classified, showing 11 genotypes based on the 5′-UTR microsatellite and 193 different tandem repeats with high amino acid variability per position. Our results showed phylogenetic correlation between MSP1a sequence, secondary structure, B-cell epitope composition and tick transmissibility of *A. marginale* strains. The analysis of MSP1a sequences provides relevant information about the biology of *A. marginale* to design vaccines with a cross-protective capacity based on MSP1a B-cell epitopes.

## Introduction

Bovine anaplasmosis, caused by the intraerythrocytic rickettsia *Anaplasma marginale* (Rickettsiales: Anaplasmataceae), is an economically important disease of cattle which is endemic in tropical and subtropical regions of the world [1], [2]. This obligate intracellular pathogen can be transmitted biologically by ticks, mechanically by transfer of infective blood on fomites or the mouthparts of biting insects [1], [2], and, less commonly, by transplacental transmission from dams to their calves [3].

Many geographic strains of *A. marginale* have been identified worldwide which differ in morphology, protein sequence, antigenic characteristics and their ability to be transmitted by ticks [1], [2], [4]–[15]. The genetic diversity of *A. marginale* strains derived from bovine erythrocytes has been characterized based on the sequence of major surface protein (MSP) genes, several of which have been shown to be involved in host cell/pathogen interactions [16]. MSP1a, one of six MSPs described previously on *A. marginale*, is a 70–100 kDa protein encoded by a single-copy gene, *msp1a*, which is conserved during the multiplication in cattle and ticks [17]. MSP1a is involved in adhesion of *A. marginale* to bovine erythrocytes and tick cells and therefore is a determinant of infection for cattle and transmission of *A. marginale* by ticks. MSP1a has also been shown to be involved in development of bovine immunity against *A. marginale* [3]. Strains of *A. marginale* were originally identified by differences in the molecular weight of MSP1a because of variable number of 23–31 amino acid serine-rich tandem repeats located in the N-terminal region of the protein which is continuous with a highly conserved C-terminal region [6], [11], [14]. Because the number and sequence of tandem repeats remained the same in a given strain, the *msp1a* gene was recognized as a stable genetic marker for geographic strain identity [9], [12], [15], [18]–[20]. Phylogenetic analyses of *A. marginale* strains using MSPs were reported by de la Fuente et al. [14], [21]–[23]. While sequence analysis of MSP4 provided phylogeographic information, MSP1a did not prove to be as suitable for these studies [24]. However, MSP1a repeat sequence analysis contributed to the understanding of the genetic diversity of *A. marginale* within specific regions, as well providing insight into the evolution of host–pathogen-vector interactions [14], [21]–[23], [25].

MSP1a also contains neutralization sensitive T- and B-cell epitopes required for development of a protective immune response [8], [10], [26]–[29]. One B-cell epitope within the MSP1a tandem repeat ((Q/E)ASTSS) was recognized by a monoclonal antibody that neutralized *A. marginale* in vitro [6]. This neutralization-sensitive epitope was found to be conserved among heterologous *A. marginale* strains [29], [30]. An additional linear B-cell epitope (SSAGGQQQESS) was found to be immuno dominant [26], [28], [31]. Cattle immunized with MSP1 were partially protected against challenge with homologous and heterologous strains [32]–[34]. Furthermore, MSP1a antibodies reduced the infectivity of *A. marginale* for cultured tick cells [35] and infection and transmission of *A. marginale* by *D. variabilis* [1].

MSP1a is relevant to many facets of *A. marginale* research. Strain classification enables a comprehensive study of the extensive worldwide diversity of *A. marginale*. As reported herein, development of an unified nomenclature of MSP1a from *A. marginale* strains based on all available sequence data allowed for review and characterization of the worldwide genetic diversity of *A. marginale*. The information generated from these studies will be fundamental toward understanding the functional and immune relevance of *A. marginale* MSP1a and in formulating vaccines that will be cross-protective among these diverse strains.

## Results and Discussion

### Classification of *A. marginale* Strains Using MSP1a Sequence Data

In this study we propose a unified nomenclature for the classification of *A. marginale* strains based on the sequences of the MSP1a tandem repeats and the 5′-UTR microsatellite. This approach was supported by the following considerations: (i) the availability of numerous *A. marginale* MSP1a sequences in GenBank, (ii) the fact that MSP1a is encoded by a single-copy gene [1], (iii) the tandem repeat structure and sequence vary among strains from different geographic locations, while the remaining portion of the protein is highly conserved [14], (iv) the tandem repeats structure is a stable genetic marker that is conserved within a strain during the acute and persistent chronic phases of the *A. marginale* infection in cattle and after passage and transmission by ticks [1], (v) the tandem repeats contain functional domains that serve as adhesins for bovine erythrocytes and tick cells, a prerequisite for infection of host cells [10], [36], (vi) the tandem repeats contain relevant B cell epitopes and neutralization epitopes important for natural or induced immune protection in cattle [6], [31], and (vii) a microsatellite which has been implicated in the regulation of MSP1a expression levels is located in the 5′-UTR of the *msp1a* gene [25].

**Figure 1:**
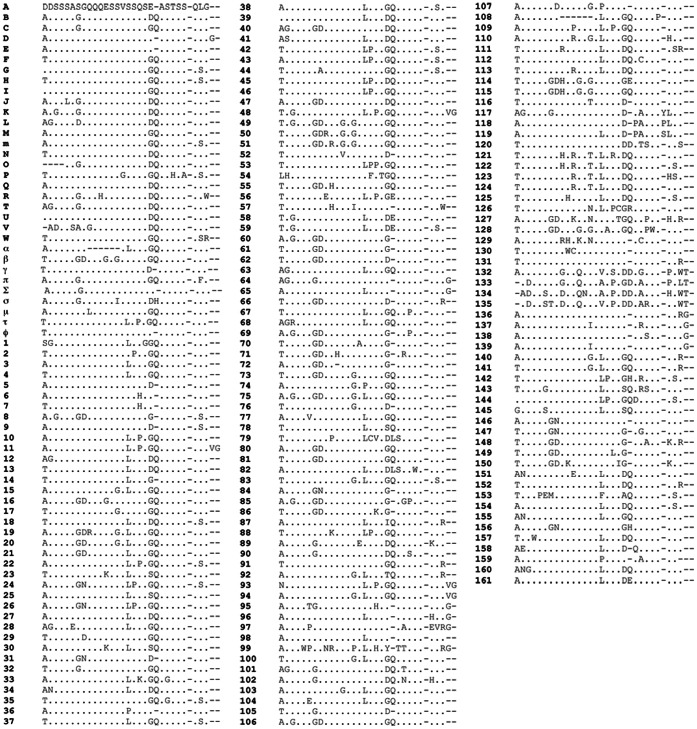
MSP1a tandem repeat sequences in A. marginale strains. The one letter amino acid code was used to depict MSP1a repeat sequences. Dots indicate identical amino acids and gaps indicate deletion/insertions. The ID of each repeat form was given following the nomenclature proposed by de la Fuente et al. (2007) [14]. The sequences from 114 until 161 are the newly classified.

**Figure 2:**
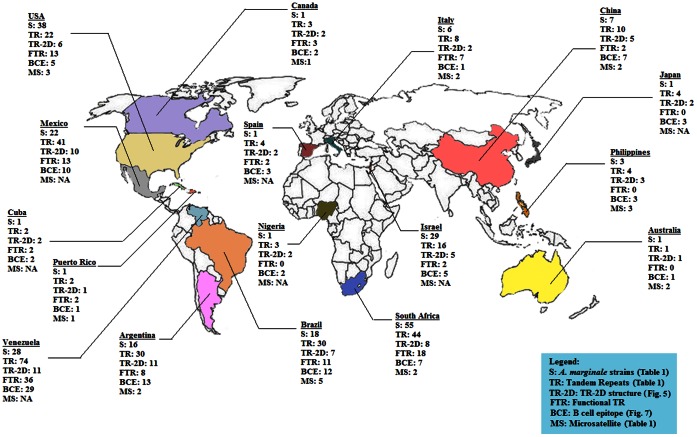
World A. marginale MSP1a molecular map. The worldwide molecular characterization of A. marginale MSP1a sequences is shown. The number of A. marginale strains (S), tandem repeats (TR), tandem repeat 2D structures (TR-2D), functional tandem repeats (FTR) containing D and E at position 20 and B cell epitope types (BCE) and microsatellites (MS) are represented for each country. Primary data is depicted in figures 1, 3 and 6. The information on 5′ UTR microsatellites is not available (NA) for some sequences.

**Figure 3:**
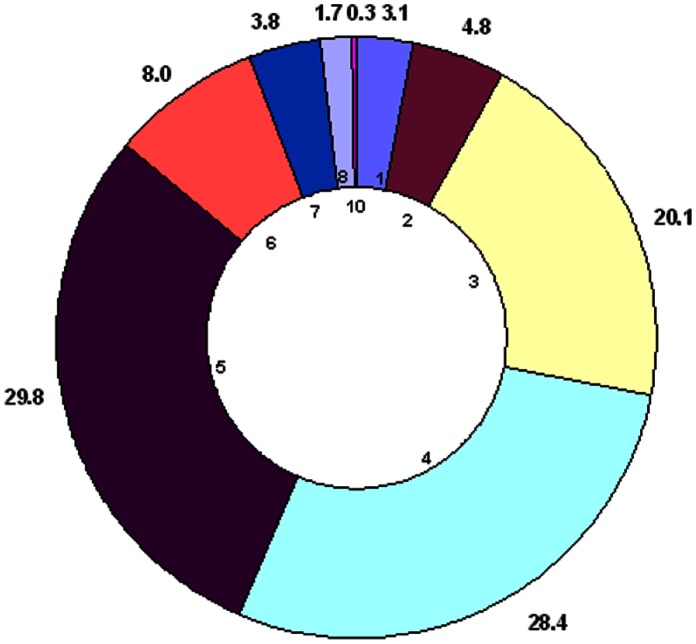
Number of tandem repeats among A. marginale strains. The total number of strains classified in our study were organized by the number of MSP1a tandem repeats. The percent of A. marginale strains (external numbers) containing different number of tandem repeats (internal numbers) is shown. The most common numbers of MSP1a tandem repeats among strains were 3 (yellow), 4 (light blue) and 5 (violet).

In this study, 193 different MSP1a tandem repeats were identified, 79 of which were published in GenBank but not formally classified (Fig. 1). Two new microsatellite structures were described in our analysis and named J and K (J: m = 1, n = 8, d = 21; K: m = 2, n = 8, d = 25) after Estrada-Peña et al. [25]. Unique *A. marginale* strains (224; 77% of all sequences found) are based on differences in geographic location, the number and structure of the MSP1a tandem repeats and microsatellites when available. These *A. marginale* strains came from 17 world regions providing a global MSP1a diversity (Fig. 2), and were classified following our proposed nomenclature (Table S1). The majority of *A. marginale* strains had more than one MSP1a tandem repeat and the maximum number of repeats was 10. No strains were reported with 9 tandem repeats (Table S1 and Fig. 3). Tables 1 provide a list of the most commonly reported strains and tandem repeats. The majority of strains were seen in only a given region, although several strains were isolated from multiple South American countries (Argentina/Chaco/− (τ, 22, 13, 18) from Argentina and Mexico; Brazil/Parana/− (τ, 10, 15) from Brazil and Argentina; Mexico/Pichucalco/E - (α, β, β, Γ^2^) from Argentina, Brazil and Mexico; and Mexico/Tamaulipas/− (64, 65, D, 65, E) from Mexico and Venezuela). The strain, Argentina/Santa Fe/− (α, β^3^, Γ), was the only strain found in more than one continent, and was reported in Argentina, Mexico, and Taiwan. Most of the MSP1a tandem repeats were shared between different strains, and repeat B, the most common tandem repeat sequence, occurred in 43 strains (Table 1). While some tandem repeats were unique to one country (repeat 72 was only reported in Brazil) or continent (repeat B was found throughout the American continent), some repeats appeared to be distributed worldwide (repeat M was reported in Israel, Italy, USA and South America). This weak association between specific tandem repeat sequences and particular geographic regions was reported previously by de la Fuente et al. [14] and may be attributed to worldwide cattle movement, among other factors. Notably, in Australia, in which introduction of cattle has been limited, only one MSP1a genotype has been reported [37].

**Table 1:** Geographical occurrence of the most common *A. marginale* strains.

Strains	Sructure of MSP1a tandem repeats	Number of strains	World occurrence
**Most common**	τ 22 13 18	7	4x Argentina, 3x Mexico
	α **β** β β Γ	7	4x Argentina, 2x Mexico, 1x Taiwan
**Second common**	34 13 4 37	6	6x South Africa
**Third common**	**B** B M	5	5x Argentina
	F **M** M	5	4x Argentina, 1x Mexico

The most frequent A. marginale strains and their geographical occurrence are shown. The most common tandem repeats found among all the A. marginale strains are underlined and there were found more than 60 (**M**), 80 (**β**) and 90 (**B**) times.

### The Biological Implications of Sequence Variation of MSP1a Tandem Repeats

The tandem repeated portion of the N-terminal region of the *A. marginale* MSP1a has been shown to be an adhesin for bovine erythrocytes and tick cells, and thus are involved in pathogen infection of host cells and transmission by ticks [10], [36], [38]. In contrast, the MSP1a N-terminal tandem repeats are absent in *A. marginale* subsp. centrale. Although *A. centrale* can be transmitted by *Rhipicephalus simus*, the tick species from which this organisms was initially isolated, this *Anaplasma* sp. cannot be transmitted by other tick species that are known to be *A. marginale* vectors [20], [39].

These analyses provided information on the range and frequency of variations in the *A. marginale* MSP1a tandem repeats. Herein, we present the sequence variation data and discuss biological implications of these findings, including O-glycosylation, amino acids at position 20 for binding to tick cell extract (TCE), protein conformation, pathogen-environmental relationships, and combination of these factors.

#### O-glycosylation

**Figure 4:**
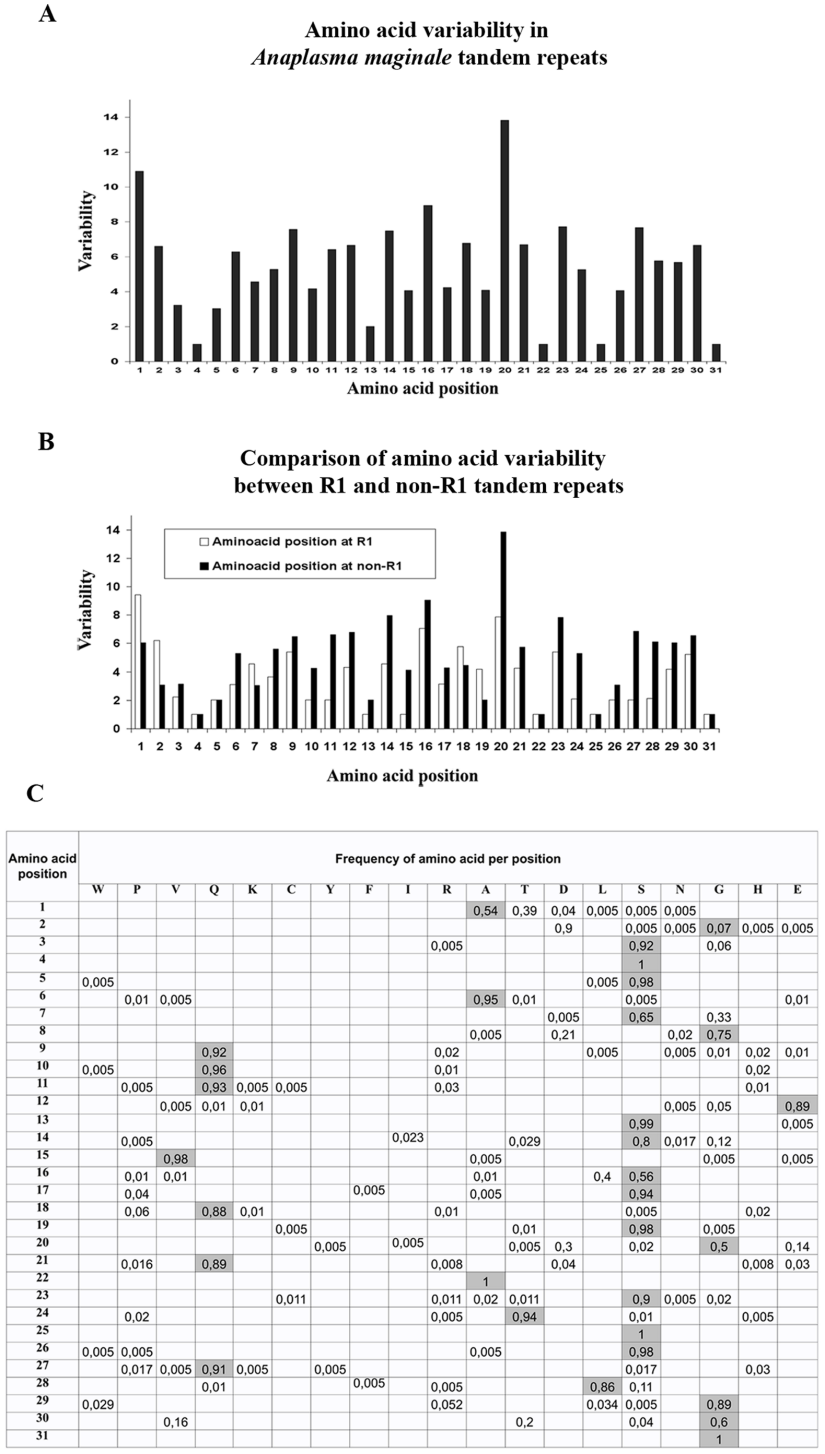
Amino acid variability and frequency in A. marginale MSP1a tandem repeats. The amino acid variability (A), comparison of the variability between tandem repeats at positions R1 and non-R1 (B) and frequency (C) were calculated per amino acid position in the MSP1a tandem repeats using the formula Variability = number of different amino acids at a given position/frequency of the most common amino acid at that position [50]. The one letter amino acid code was used to name the amino acids in (C) and the most frequent amino acids per position are colored in gray.

MSP1a tandem repeats were found to have a high variability across almost all the 31 amino acid positions, suggesting considerable evolutionary pressure on this molecule (Fig. 4A). Four positions were totally conserved: serine (S)4 and S25, alanine (A)22 and Glicine 31 (Fig. 4A). MSP1a has been shown to be O-glycosylated, with S/threonine (T) regions present in the tandem repeats as the target site for this type of glycosylation [31]. Furthermore, the binding capacity of MSP1a to tick cells diminished after deglycosylation [31]. The conservation of S4 and S25 among all the tandem repeats included in this study could indicate that the O-glycosylation at these two positions is highly relevant for *A. marginale* infection. Several bacterial glycoproteins have also been reported to play a role in bacterial adhesion, invasion and pathogenesis [40].

#### Relevance of amino acids at position 20 for binding to tick cell extract (TCE)

Within the MSP1a tandem repeats, the negatively charged amino acids, aspartic acid (D) and glutamic acid (E), at position 20 were shown to be essential for binding of MSP1a to TCE. When glycine (G) was located at position 20, binding was not observed [10]. This result suggested that the amino acid at position 20 may be essential for *A. marginale* binding to tick cells, a prerequisite for pathogen infection and transmission by ticks. In fact, previous experiments confirmed the existence of both tick-transmissible and not transmissible *A. marginale* strains and, at least for some strains, the presence of TCE-binding with tandem repeats correlated with strains that were transmissible by *Dermacentor sp*. ticks [10]. In all strains, the first MSP1a tandem repeat (R1) contained 67 (34.7%) different sequences. However, R1 tandem repeats had less amino acid variability and 6 conserved positions when compared to non-R1 tandem repeats, in which only 4 conserved amino acid positions were found (Fig. 4B). These results suggested that the R1 tandem repeat may play a role in *A. marginale* infection and transmission. We found 87 tandem repeats containing D20 (71%) or E20 (29%) (Fig. 1). In total, 161 *A. marginale* strains contained one of these tandem repeats at least once and in 114 (71%) of these strains, the D20 or E20 was found in the R1 tandem repeat. Surprisingly, the highest variable amino acid was at position 20 (Fig. 4A), suggesting greater evolutionary pressure at this amino acid position. From our findings, G was the most frequent amino acid at position 20 (Fig. 4C), in both R1 and non-R1 tandem repeats (data not shown), but only 4 amino acids were found at position 20 in R1 (from highest to lowest frequency: G, D, E and serine [S]) while 7 different amino acids were found at position 20 in non-R1 tandem repeats (G, D, E, S, T, isoleucine [I] and tyrosine [Y]) (Fig. 4C). In previous experiments, non-R1 tandem repeats had a phylogenetic correlation with tick-transmissible strains, but this correlation was not seen with R1 tandem repeats [9]. We propose that non-R1 tandems are also involved in *A. marginale*-tick interactions which require more genetic variability, because more than 20 different tick species have been reported to transmit *A. marginale* [24].

#### Protein conformation

**Figure 5:**
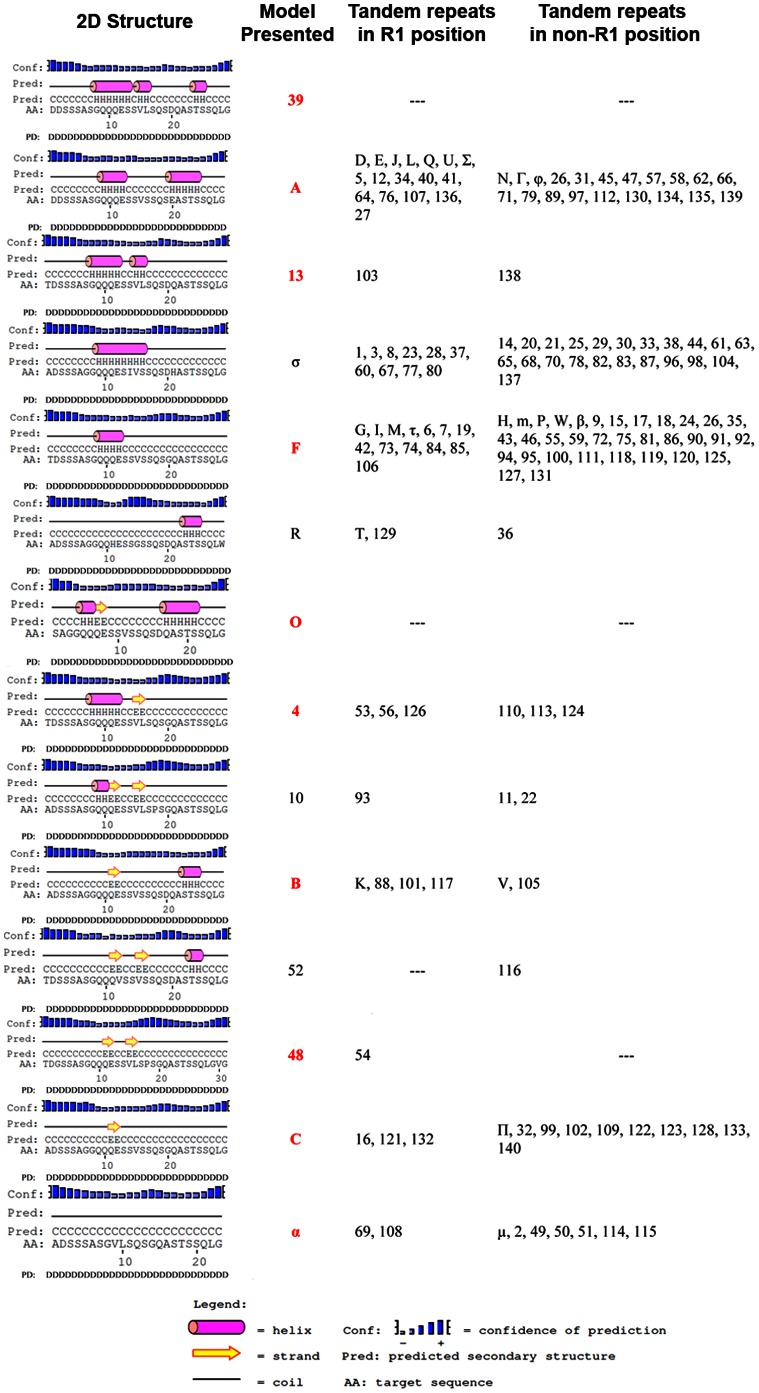
Changes in putative 2D structure and disorder analysis of A. marginale MSP1a tandem repeats. The PSIPRED web server was used to predict the 2D structure. The tandem repeats were grouped into fourteen 2D structure models. Tandem repeats shown represent prototypes of corresponding tandem repeat 2D structures. The second column shows (model presented) the ID of the tandem repeat presented as prototype. Models ID in red represent tandem repeats in R1 position (first tandem in the MSP1a sequence).

As proposed previously both amino acid sequence and protein conformation may contribute to the function of MSP1a as adhesin [10]. Herein, we explored this hypothesis by predicting the 2-D structure of all the MSP1a tandem repeats. We found that 14 models explained all of the variability of 2-D structure among the 193 tandem repeats (Fig. 5). Three α-helical 2-D structure models, differing in the length and amount of α helixes in the tandem repeat, described 68% of the 2-D structure variation (presented as A, σ and F in Fig. 5). The analysis revealed that the amino acid at position 20 correlated with specific 2-D structure changes in the tandem repeat. When D or E amino acids were at this position, the structure of the tandem repeat was predominantly long α-helical structures (Model types 39, A, 13 and σ), but when a G was in this position, the repeat was a short α helix, β-strand or coiled 2-D structure (Model types 4, 10, α and 48) (Fig. 5). The other four amino acids that were found at lower frequencies at position 20, (I, Y, T and S; Fig. 4C), except for Y, retained the α-helical 2-D structure (Fig. 5).

Our results suggest that the MSP1a tandem repeat 2-D structure also correlated with tick transmissibility (Table 2). Strains reported previously that were not transmitted by *Dermacentor sp.* had a predominant pattern for 2-D structure of tandem repeats of β strand, short α-helix or coiled structures, regardless of whether or not they had TCE-binding tandem repeats (Table 2). In contrast, abundant α-helices were found in tandem repeats of strains transmitted by ticks (Table 2). In the last case, as shown for the USA/Florida/G - (A, B^7^) strain, the presence of all seven TCE-binding tandem repeats did not correlate with tick-transmissibility; this Florida isolate was clearly shown to be non-infective for ticks or cultured tick cells (Table 2). However, the 2-D structure appeared to be a determinant for the biological transmission of *A. marginale*, because the Israel/Israel tailed/F - (1, F, M, 3) strains, while not having TCE-binding repeats but did have α-helices as 2-D structure, were tick transmissible (Table 2). As listed in Table 2, the data collected thus far regarding *A. marginale* transmissibility by ticks is related to the major vector *Dermacentor sp.* The complexity of the relationship between the 2-D structure, TCE-binding repeats and tick transmissibility was also seen with the Brazil/Minas Gerais/E strain–(13, 42, 13, 18) which does not contain β strands and is not transmissible by *Rhipicephalus (Boophilus) microplus* [13]. This example demonstrated a different pattern as that observed with *A. marginale* that are not transmissible by *Dermacentor sp.* The 2-D structure data presented in the present study is in agreement with an analysis performed recently on *A. marginale* MSP2 variants in tick or mammalian cells [41]. The 2-D structure analysis using PSIPRED demonstrated that MSP2 variants expressed in ticks were predominantly α-helices, while β-strands were present in MSP2 variants expressed only in mammalian cells [41], [42].

**Table 2:** Effect of putative MSP1a tandem repeat 2-D structure on A. marginale tick transmission phenotype.

Strains	MSP1a tandem repeats 2D structure	Transmission by ticks		
		Dermacentor spp.	R. sanguineus orR. microplus	H. excavatum
USA/Idaho/C - (D^5^, E)	(α-α, α-α, α-α, α-α, α-α, α-α)	Yes ^(*)^	ND	ND
Puerto Rico/Puerto Rico/C - (E, φ^5^)	(α-α, α-α, α-α, α-α, α-α, α-α)	Yes ^(***)^	Yes ^(***)^	ND
USA/Virginia/G - (A, B)	(α-α, β-α)	Yes ^(*)^	ND	ND
USA/St.Maries/G - (J, B^2^)	(α-α, β-α, β-α)	Yes ^(*)^	Yes^(***)^	ND
USA/Oklahoma/G - (U)	(α-α)	Yes^(+)^	ND	ND
USA/Missisippi/D - (D^4^, E)	(α-α, α-α, α-α, α-α, α-α)	Yes^(*)^	ND	ND
USA/Rassmusen/− (A, F, H)	(α-α, α-c, α-c)	Yes^(*)^	ND	ND
USA/Kansas/− (E, M, φ)	(α-α, α-c, α-α)	Yes^(−)^	ND	ND
Nigeria/Zaria/− (54, 55, F)	(β-β, α-c, α-c)	Yes^(**)^	ND	ND
Israel/Israel tailed/F - (1, F, M, 3)	(α-c, α-c, α-c, α-c)	ND	Yes^(****)^	Yes^(****)^
Israel/Israel non tailed/G - (1, 4)	(α-c, α-β)	ND	Yes^(****)^	No^(****)^
USA/Florida/G - (A, B^7^)	(α-α, β-α, β-α, β-α, β-α, β-α, β-α, β-α)	No^(*)^	ND	ND
USA/California/G - (B^2^, C)	(β-α, β-α, β-c)	No^(*)^	ND	ND
USA/Okeechobee/G - (L, B, C, B, C )	(α-α, β-α, β-c, β-α, β-c)	No^(*)^	ND	ND
USA/Illinois/G - (M, N, B, M, H)	(α-c, α-α, β-α, α-c, α-c)	No^(*)^	ND	ND

The information about transmission of A. marginale strains by ticks was collected from (*) de la Fuente et al. (2003) [10],

(**) Zivkovic et al. (2007) [65],

(***) Futse et al. (2003) [44],

(****) Shkap et al. 2009 (****) [39],

(−) Leverich et al. (2008) [66], and (+) Barbet et al (2001) [67].

TCE-binding tandem repeats are underlined. Abbreviation: ND, not determined.

#### Pathogen-environmental relationships

*A. marginale* was recorded in four eco-region clusters defined in our study (Table 3). Eco-region Cluster 1 extended over large areas of central Africa and central South America, primarily Argentina and southern Brazil, and was a region with medium to high Normalized Difference Vegetation Index (NDVI) values and a well-defined seasonal decrease between June and September. The highest recorded temperature and annual rainfall of approximately 1,000 mm occurs in Eco-region Cluster 1. Eco-region Cluster 2 included vast areas of the Mesoamerican corridor, northern South America and a small territory of eastern South Africa, and included zones with high NDVI throughout the year without seasonal variability. The temperature values in Eco-region Cluster 2 were similar to those in Eco-region Cluster 1, but with an annual rainfall of approximately 1,500 mm. Eco-region Cluster 3 extended over central South Africa and scattered parts of the southern USA and Mexico, and had the lowest NDVI values with minimal change across the year. This eco-region had lower temperature values and minimum rainfall. Finally, Eco-region Cluster 4 extended over large areas of the USA and had a clear NDVI signature that was low between November and March and then rose to maximum levels in July. This area was the coldest among the four eco-region clusters, with an annual rainfall of approximately 800 mm/year. The results of this study demonstrated that 82% of MSP1a R1 unique sequences were associated with only one eco-region cluster (Table 3). Seventeen R1 unique sequences (27% of the total number of R1 sequences) were reported exclusively in Eco-region Cluster 1 and shared 16 out of 31 amino acids (51.6% of the total number of amino acids) (Table 3). Sixteen R1 unique sequences (17%) were reported only in Eco-region Cluster 2 which had 64.5% identical amino acids (Table 3). Twenty-five R1 unique sequences (32%) were only found in Eco-region Cluster 3, of which 64.5% of their amino acids were shared (Table 3). Only five R1 sequences were exclusively associated with Eco-region Cluster 4, which had 77.4% identical amino acids (Table 3). Eight R1 sequences, were found simultaneously in more than one of the eco-region clusters (Table 3). These results confirmed that *A. marginale* MSP1a R1 sequences clustered according to a pattern of abiotic (climate) factors, and are related to both the species of tick vector and the performance of this tick vector in the eco-region [25]. Higher variability in R1 repeat sequences appeared in areas where several tick species are candidate vectors (i.e. USA and Canada) or where mechanical transmission is common (i.e. central Argentina). Remarkably, only one *A. marginale* MSP1a genotype has been recorded in Australia (Table S1) along with a single tick vector species, *Rhipicephalus australis* [43]. As reported previously, the hypothesis of strain geographic association was rejected [25]. Mantel's test on R1 sequences was 0.82 (P<0.001) when applied to eco-region clusters using only unique sequences. The same test provided a value of 0.31 (P = 0.145) for the distances matrix based on geographical association of strains. All the *A. marginale* MSP1a R1 sequences within each eco-region cluster appeared to be under positive selection as shown by dN/dS indexes of 1.83, 1.61, 1.54 and 1.21 for Eco-region Clusters 1 to 4, respectively. Therefore, these results confirmed the hypothesis that *A. marginale* strains are associated with factors that drive the biological performance of ticks vectors in each region [25].

**Table 3:** Association of *A. marginale* MSP1a R1 sequences with world ecoregions.

Ecoregion	R1 sequences(a)	Other R1 sequences(b)
1: central Africa and central South America, primarily Argentina and southern Brazil	M, 4, 8, 12, 16, 56, 60, 64, 67, 69, 72, 78, 93, 132, γ, π, τ	A, B, D, T, 13, 23, α
2: Mesoamerican corridor, northern South Americaand a small territory of eastern South Africa	E, F, 28, 37, 48, 53, 54, 84, 85, 101, 117, 121,126, 129, 136, ε	A, B, L, T, 13, 23, α
3: central South Africa and scattered parts ofsouthern USA and Mexico	M, O, Q, U, 1, 3, 5, 6, 7, 27, 33, 34, 39, 40, 42, 74,77, 82, 141, 142, 143, 147, 151, 154, 155,	A, D
4: USA	I, J, K, O, U, 19,	A, B, L, α

World ecoregions were built upon temporal series of NDVI values.

(a) R1 sequences recorded in one ecoregion only.

(b) R1 sequences that have been reported in other ecoregions.

#### Influence of a combination of factors

**Figure 6:**
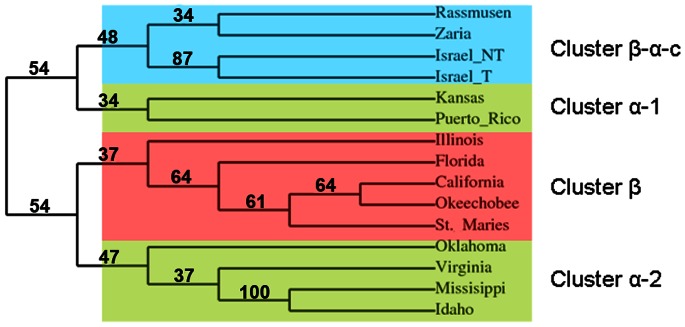
Phylogenetic tree based on MSP1a tandem repeat amino acid sequences. The MSP1a sequences from tick-transmissible and non-transmissible strains (Table 2) were included in the phylogenetic analysis. The phylogenetic tree was reconstructed using the neighbor joining and maximum likelihood methods. Reliability for internal branch was assessed using the bootstrapping method with 1000 bootstrap replicates. Bootstrap values are shown as % in the internal branch. The tree shows four phylogenetic clusters containing different patterns of MSP1a tandem repeat 2D structures. Cluster β-α-c (blue), cluster α-1 and cluster α-2 (beige) contain tick-transmissible A. marginale strains while in cluster β (red) fall the non-tick-transmissible strains.

A phylogenetic correlation was found among *A. marginale* strains between MSP1a tandem repeats 2-D structure, transmissibility by ticks and the presence of TCE-binding tandem repeats (Fig. 6). Notably, cluster β contains all non-tick-transmissible *A. marginale* strains, abundant β-strand tandem repeat 2-D structure, and a low proportion of TCE-binding repeats (Fig. 6). The exception to this rule is the USA/St. Maries/G – (J, B^2^) strain, which is tick-transmissible [34], [44] but falls into this cluster. This position of the USA/St. Maries/G – (J, B^2^) strain in the phylogenetic tree suggests that *A. marginale* tick-transmissible strains may evolve from non-tick-transmissible strains. The cluster α-2 contains tick-transmissible strains with the highest proportion of α-helices and all TCE-binding tandem repeats. In contrast, strains in cluster β-α-c have a more variable 2-D structure and a high proportion of TC non-binding tandem repeats. The high β-strand content and short α-helixes in MSP1a tandem repeats appears to be associated with a non-tick-transmissible phenotype, similar to the results reported recently with MSP2 sequence study [41]. However, variable 2-D structures such as those in cluster β-α-c may be required in order to bypass the absence of TCE-binding tandem repeats and maintain the tick-transmission phenotype. The presence of TCE-binding tandem repeats could contribute to the organization of the MSP1a molecule, as seen in cluster α-1, where high content of α-helices correlated only with the presence of TCE-binding tandem repeats. Additionally, the analysis using the GeneSilico Metaserver predicted that tandem repeats have a protein disorder across the whole tandem repeat (data not shown). Intrinsically disordered proteins demonstrated better molecular recognition due to a higher specificity, larger interacting surfaces and different folding patterns upon binding [45].

### Analysis of B Cell Epitope in MSP1a Tandem Repeats

Variation in *A. marginale* outer membrane proteins, such as MSP1a, is a major challenge in developing vaccines that can provide cross-protection between the diversity of strains worldwide. MSP1a has long been investigated as a vaccine candidate [68], [32]–[34] due to the presence of a conserved neutralization-sensitive B-cell epitope at position 20–26 of tandem repeats [6], [29]. However, a study [31] of the the antibody response to the strain USA/Oklahoma/G - (K, C, H), demonstrated that after vaccination with whole *A. marginale* or recombinant MSP1a, a different MSP1a B-cell epitope was immunodominant, SSAGGQQQESS, a linear epitope at amino acid positions 4 to 14 of the tandem repeat. As the antibody response is of principal importance in anaplasmosis, strain to strain variation in tandem repeat B-cell epitopes would be an important consideration in development of an MSP1a recombinant vaccine [46]–[48]. We therefore characterized the diversity of the immunodominant position 4–14 B-cell epitope among sequenced strains.

**Figure 7:**
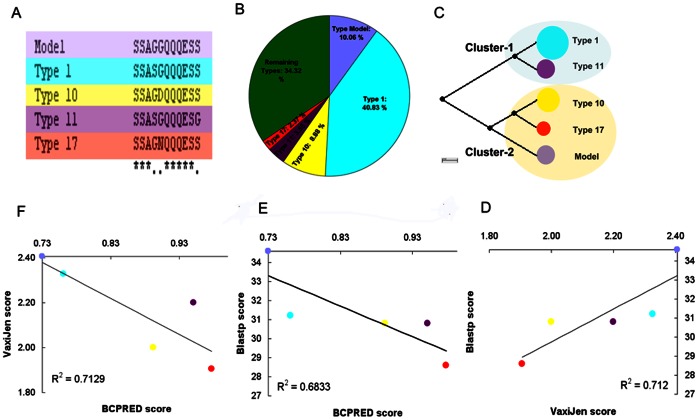
B-cell epitope analysis in A. marginale MSP1a tandem repeats. The B-cell epitopes were predicted using BCPRED server. The type 1 B-cell epitope was used as reference (Model) for comparisons. (A) Clustalw alignment and amino acid changes in the 5 more represented MSP1a tandem repeat B cell epitopes. B-cell epitope types model (light violet), 1 (blue), 10 (yellow), 11 (dark violet) and 17 (red) are shown. (B) Percent of tandem repeats containing each type of B cell epitopes. (C) Neighbor joining phylogenetic tree based on B cell epitope amino acid sequences showing the two clusters formed by the 5 more represented B cell epitopes. Cluster-1: Types 1 and 11 and Cluster-2: Types Model, 10 and 17. Correlations between VaxiJen/Blastp (D), BCPRED/Blastp scores (E) and VaxiJen/BCPRED (F) scores are shown. These correlations suggest that the epitopes with higher homology (Blastp score) share in common the immunogenic properties represented by VaxiJen/BCPRED.

This epitope showed high sequence variability among all MSP1a sequences reported to date (Fig. 4A). From the 172 MSP1a tandem repeats included in the B-cell epitope analysis, 53 sequence variants were found; nevertheless 5 of those variants covered 64% of the total epitope variability (Figs. 7A and 7B). These 5 variants formed 2 phylogenetic clusters (Fig. 7C); variants in cluster 2 share the same antibody recognition site, while those in cluster 1, types 1 and 11, have different antibody recognition sites (data not shown). All B-cell epitope types were surface exposed (data not shown) as was previously predicted for the Type 1 B-cell epitope using the TMHMM2 algorithm [31].

Seven of the 53 B-cell epitope variants gave a 0 score in both B-cell epitope prediction servers BCEPRED and BCPREDS (data not shown), suggesting that some amino acid changes in the immunodominant B-cell epitope (amino acids 4–14) could be the determining factor for the loss of this epitope. Analysis by VaxiJen, a predictor of protective antigens [49], demonstrated that the highest VaxiJen score belongs to the type model B-cell epitope, while types 1, 10, 11 and 17 have VaxiJen scores lower than the type model but higher than the average for all 53 epitopes (Fig. 7D). Among the main types of B-cell epitopes, a linear but negative correlation was observed between VaxiJen and BCPREDS scores and between Blastp and BCPREDS scores (Figs. 7E and 7F), suggesting a relationship between sequence identity and immune properties among the B-cell epitopes. Overall, these results suggested that different immune properties exist among the different MSP1a types of the B-cell epitopes.

As this is an immunodominant epitope [31], tandem repeats with epitopes predicted to be recognised by different antibodies could be a factor in the frequent lack of cross-protection between heterologous strains. Conversely, strains which share the same type of antibody recognition site may be more likely to be cross-protective.

A correlation (R^2^ = 0.69) was found between the number of 2-D structure models present in a given geographic location and the amount of B-cell epitope types in the same region (Fig. 2). Therefore, we explored the hypothesis that there was a link between 2-D structure and B-cell epitopes among the MSP1a tandem repeats. An α-helical structure was seen in 88% of the tandem repeats containing type 1 B-cell epitopes and in 100% of tandem repeats containing types 10, 11 or 17 B-cell epitopes. In contrast, 69% of the tandem repeats containing type model B-cell epitopes had β-strand structures. Interestingly, a correlation was found between tick transmissibility and the type of B-cell epitopes present on MSP1a repeats, possibly due to these structural differences between epitope types. 71% of the MSP1a tandem repeats present in non-tick-transmissible *A. marginale* strains were found to have type model B-cell epitopes, whereas 87% of the tandem repeats in tick transmissible strains contained type 1 B-cell epitopes. This data suggest antigenic differences between tick-transmissible and not-transmissible *A. marginale* strains, and agrees with the finding that both type 1 and model type epitopes fall into different phylogenetic clusters (Fig. 7C) presenting different putative antibodies recognition sites. Both epitopes had the highest VaxiJen and BCPRED scores among the 5 most common B-cell epitopes, but shared low identity as shown by Blastp score (data not shown).

Collectively, the results of these studies demonstrate that the unified nomenclature proposed herein using MSP1a sequences provides information about *A. marginale* strain world distribution, transmissibility by ticks, infective potential, antigenic variability and putative utility for MSP1a vaccine development. The structural and immune analyses of MSP1a revealed a phylogenetic correlation between A. marginale tick transmissibility, 2-D structure adopted by the tandem repeats and the type of B-cell epitopes present in the tandem repeats. These results are fundamental information for design of MSP1a structure-based vaccines which would be cross protective against multiple *A. marginale* strains, and for development of serodiagnostic methods based on differential B-cell epitopes, for epidemiological characterization of field strains.

## Methods

### *Anaplasma marginale* Strains Classification

A total of 289 A. marginale MSP1a sequences with complete tandem repeat regions included in this study were obtained from published research and the GenBank sequence database [http://www.ncbi.nlm.nih.gov/]. These sequences were analyzed and classified, and the tandem repeats were named (or renamed) following the nomenclature proposed by Allred et al. [6] and de la Fuente et al. [14]. When microsatellite sequences were included in the *msp1a* published nucleotide sequence, they were used to assign a genotype following the system of Estrada-Peña et al. [25]. Briefly, the 5′-UTR microsatellite located between the putative Shine-Dalgarno (SD) sequence (GTAGG) and the translation initiation codon (ATG), GTAGG (**G/A TTT**) m (**GT**) n **T** ATG (microsatellite sequence is shown in bold letters) and the SD-ATG distance (d) calculated in nucleotides as (4 × m)+(2 × n) +1 were used. We propose one nomenclature for *A. marginale* strains based on MSP1a with the following structure: country/locality/microsatellite genotype - (structure of tandem repeat), and all MSP1a sequences were classified using this nomenclature. When multiple strains had 100% amino acid sequence similarity across tandem repeats, they were listed under one strain name, with geographical information taken from the isolate with the most complete information. When this information was equal between isolates, information was used from the isolate first submitted to GenBank.

### Amino Acid Variability within MSP1a Tandem Repeats

Tandem repeat sequences were aligned using Clustalw, and each amino acid position was numbered from 1 to 31. The amino acid variability was determined using the formula of Kuby et al. [50]. The variability was equal to the number of different amino acids at a given position/frequency of the most common amino acid at that position.

### Correlation Analysis between MSP1a Tandem Repeats and World Ecological Regions

The analysis was conducted as described previously, assuming that (i) eco-regions could be delineated by quantitative abiotic characters based on well-recognized and repeatable attributes and (ii) *A. marginale* strains were associated with each eco-region and subjected to different environmental conditions that could be analyzed by multivariate geographic clustering [25]. The feature selected to build the eco-regions was the NDVI, which is a variable that reflects vegetation stress and summarizes information about the ecological background for the performance of tick populations [25]. A 0.1° resolution series of monthly NDVI data was obtained for the period 1986–2006. The 12 averaged monthly images were subjected to Principal Components Analysis (PCA) to obtain decomposition into the main axes representing the most significant, non-redundant information. The strongest principal axes were chosen using Cattell's Scree Test [25]. The PCA analysis retained three principal axes, including 92% of the total variance. A hierarchical agglomerative clustering on PCA values was then used to classify multiple geographical areas into a single common set of discrete regions. Mahalanobis distance was used as a measure of dissimilarity and the weighted pair-group average was used as the amalgamation method. A value of 0.05 was used as the cut-off probability for assignment to a given eco-region.

### Bioinformatics

Secondary structure was predicted using the position-specific scoring matrices method [51] from the PSIPRED server [52], and protein disorder was predicted using the GeneSilico Metaserver [53].

The immunodominant B-cell epitope SSAGGQQQESS (amino acid positions 4–14), previously mapped in the A. marginale strain USA/Oklahoma/G - (K, C, H) MSP1a sequence [31] will be referred to as epitope “Type 1″. The variability among MSP1a tandem repeats within this B-cell epitope (amino acid positions 4–14) was evaluated. The percent of amino acid identity and Blastp score among the B-cell epitopes had a linear correlation (R^2^ = 0.85), so the Blastp score was used as an identity index in the analysis. Prediction/score of B-cell epitope was determined using BCPREDS server [54] and the protective potential of the B-cell epitope was predicted using the VaxiJen server [55]. Prediction of physicochemical properties of the B-cell epitope was assayed using BCEPRED server [56]. PepSurf algorithm [57], implemented in the PEPITOPE server [58], was used to determine the structure/position of the affinity-selected B-cell epitopes in a model protein. The 3D analysis of MSP1a tandem repeat B-cell epitopes was performed using a model of the crystal structure of the Fv corresponding with the anti-blood group A antibody AC1001 (PDB ID: 1JV5) [59].

For phylogenetic analysis, sequences were aligned with MUSCLE (v3.7) configured for the highest accuracy [60]. After alignment, ambiguous regions (i.e., containing gaps and/or poorly aligned) were removed with Gblocks (v0.91b) [61]. The phylogenetic tree was reconstructed using the neighbor joining (NJ) and maximum likelihood methods implemented in PHYLIP package (v3.66), NJ distances were calculated using FastDist [62], [63]. Reliability for internal branch was assessed using the bootstrapping method (1000 bootstrap replicates). Graphical representation and editing of the phylogenetic tree were performed with TreeDyn (v198.3) [64].

## Supporting Information

Table S1 **Classification of** ***A. marginale*** **strains based on the proposed nomenclature.** A total of 289 MSP1a sequences were analyzed. A. marginale 224 unique strains were classified using the nomenclature proposed in our study: Country/Locality/microsatellite genotype - (structure of tandem repeat). The 5′UTR microsatellite genotype was included when available. The structure of tandem repeats was represented following the nomenclature previously proposed [14] (Fig. 1). When the same repeat was present more than one time, a super-index was used to represent copy number for this repeat.
(PDF)
