# Supplementary material for: Functional and Immunological Relevance of Anaplasma marginale Major Surface Protein 1a Sequence and Structural Analysis
Source: PLoS One. 2013 Jun 11;8(6):e65243. doi: 10.1371/journal.pone.0065243 (PMC3679145; doi:10.1371/journal.pone.0065243)
Supplement: Table S1 — Classification of A. marginale strains based on the proposed nomenclature. A total of 289 MSP1a sequences were analyzed. A. marginale 224 unique strains were classified using the nomenclature proposed in our study: Country/Locality/microsatellite genotype - (structure of tandem repeat). The 5′UTR microsatellite genotype was included when available. The structure of tandem repeats was represented following the nomenclature previously proposed [14] (Fig. 1). When the same repeat was present more than one time, a super-index was used to represent copy number for this repeat. (PDF) [file pone.0065243.s001.pdf]

Strain Name	Accession No. / Reference	Tandem repeat structure							No. Tandem repeats
Argentina/Chaco/ - (α, β, Γ^2, β^2 ,Γ)	DQ833264	α	β	Γ	Γ	β	β	Γ	7
Argentina/Chaco/ - (τ, 11, 10^2, 11, 10, 15)	DQ833266	τ	11	10	10	11	10	15	7
Argentina/Chaco/ - (τ, 22, 13, 18)	DQ833263	τ	22	13	18				4
Argentina/Cordoba/ - (23, 24, 25, 26, 27^2)	DQ833261	23	24	25	26	27	27		6
Argentina/Corrientes/ - (53, 10^2)	DQ833273	53	10	10					3
Argentina/Corrientes/ - (48, 11, 10)	DQ833274	48	11	10					3
Argentina/Corrientes/ - (α, 49, 50, 51, 52)	DQ833275	α	49	50	51	52			5
Argentina/Corrientes/ - (α, β^3)	DQ833272	α	β	β	β				4
Argentina/Entre Rios/ - (F, M^2)	DQ833249	F	M	M					3
Argentina/Mercedes/ - (23, 30, 31^3)	DQ833271	23	30	31	31	31			5
Argentina/Quitilipi/ - (28, 29, M, 29, M, F)	DQ833270	28	29	M	29	M	F		6
Argentina/Salta/ G - (B^2, M)	AF428093	B	B	M					3
Argentina/Santa Fe/ - (α, β^3, Γ)	DQ833253	α	β	β	β	Γ			5
Argentina/Virasoro/E - (Σ, B, Q, B, C)	AF428094	Σ	B	Q	B	C			5
Australia/Northern Territory/J - (8)	AF407542	8							1
Australia/Western Australia/ E - (8)	AF407545	8							1
Brazil/Minas Gerais/ B - (13, 27^3)	JX844209	13	27	27	27				4
Brazil/Minas Gerais/ C - (α, β, τ, M)	AY283199	α	β	τ	M				4
Brazil/Minas Gerais/ D - (72, 62, 61)	JX844216	72	62	61					3
Brazil/Minas Gerais/ D - (C, F, N)	AY283198	C	F	N					3
Brazil/Minas Gerais/ E - (13, 159^2)	Bastos et al.2010	13	159	159					3
Brazil/Minas Gerais/ E - (72, 62, 61)	JX844210	72	62	61					3
Brazil/Minas Gerais/ E - (78, 24^2,25, 31)	JX844206	78	24	24	25	31			5
Brazil/Minas Gerais/ E - (α, β^2, 13)	JX844215	α	β	β	13				4
Brazil/Minas Gerais/ E - (α, β^2, N)	AY283200	α	β	β	N				4

Brazil/Minas Gerais/ E - (α, β^2, φ)	JX844207	**α**	**β**	**β**	**φ**				4
Brazil/Minas Gerais/ E - (τ, 57, 13, 18)	JX844213	**τ**	**57**	**13**	**18**				4
Brazil/Minas Gerais/ E - (τ, 57, β^2, φ)	JX844205	**τ**	**57**	**β**	**β**	**φ**			5
Brazil/Minas Gerais/ E - (13, 27^2)	EU676175	**13**	**27**	**27**					3
Brazil/Minas Gerais/ E - (13, 42, 13, 18)	EU676176	**13**	**42**	**13**	**18**				4
Brazil/Minas Gerais/ G - (B, Q, B, M)	JX844208	**B**	**Q**	**B**	**M**				4
Brazil/Minas Gerais/ G - (B^2,Q, σ, μ)	AF428092	**B**	**B**	**Q**	**σ**	**μ**			5
Brazil/Parana/ - (τ, 10, 15)	AY998121	**τ**	**10**	**15**					3
Brazil/Parana/ E - (16, F, 17, 13, 18)	AY998120	**16**	**F**	**17**	**13**	**18**			5
Canada/Saskatchewan/ E - (D, Q^2, R)	AY253141	**D**	**Q**	**Q**	**R**				4
China/HBA8/ - (19, 20, 19, 21)	DQ811774	**19**	**20**	**19**	**21**				4
Cuba/Habana/ - (A, B^4)	AY489564	**A**	**B**	**B**	**B**	**B**			5
Israel/ Israel round / - (1, F, M, 3^2)	AY355282	**1**	**F**	**M**	**3**	**3**			5
Israel/Golan Heights/ - (1, 4, 3^3)	EU678758	**1**	**4**	**3**	**3**	**3**			5
Israel/Golan Heights/ - (1, F, M, 3^3)	Molad et al, 2009	**1**	**F**	**M**	**3**	**3**	**3**		6
Israel/Golan Heights/ - (1, F, M^2, 3)	Molad et al, 2009	**1**	**F**	**M**	**M**	**3**			5
Israel/Golan Heights/ - (1, F, M^2, 3^2)	Molad et al, 2009	**1**	**F**	**M**	**M**	**3**	**3**		6
Israel/Golan Heights/ - (3, M, 3^3)	Molad et al, 2009	**3**	**M**	**3**	**3**	**3**			5
Israel/Golan Heights/ - (74^3, 73^2)	EU678765	**74**	**74**	**74**	**73**	**73**			5
Israel/Golan Heights/ - (74^3, 76)	EU678756	**74**	**74**	**74**	**76**				4
Israel/Golan Heights/ - (77, 25, 3^2)	EU678755	**77**	**25**	**3**	**3**				4
Israel/Golan Heights/ - (77, 25, 3^4)	Molad et al, 2009	**77**	**25**	**3**	**3**	**3**	**3**		6
Israel/Golan Heights/ - (77, 25^2, 3^2)	Molad et al, 2009	**77**	**25**	**25**	**3**	**3**			5
Israel/Golan Heights/ - (77, 3, 4, 3)	Molad et al, 2009	**77**	**3**	**4**	**3**				4
Israel/Golan Heights/ - (77, 3^4)	EU678757	**77**	**3**	**3**	**3**	**3**			5
Israel/Israel non tailed/ G - (1, 4)	AF352559	**1**	**4**						2
Israel/Israel tailed/ - (1, 2, M, 3)	AY295077	**1**	**2**	**M**	**3**				4
Israel/Israel tailed/ F - (1, F, M, 3)	AF352560	**1**	**F**	**M**	**3**				4
Israel/Jerusalem/ - (77, 3^6)	Molad et al, 2009	**77**	**3**	**3**	**3**	**3**	**3**	**3**	7

Israel/Jerusalem/ - (78, 3^2)	Molad et al, 2009	78	3	3						3
Israel/Jerusalem/ - (78, 3^3)	EU678759	78	3	3	3					4
Israel/Or-Haner/ - (M, F^2)	AY355284	M	F	F						3
Israel/Upper Galilee/ - (7, E^2, M, 3^2)	EU678761	7	E	E	M	3	3			6
Israel/Upper Galilee/ - (7, E^4)	EU678760	7	E	E	E	E				5
Israel/Upper Galilee/ - (M, F^3)	AY846868	M	F	F	F					4
Israel/Western Galilee/ - (4, 3, 4, 3)	Molad et al, 2009	4	3	4	3					4
Israel/Western Galilee/ - (74^2, 76)	Molad et al, 2009	74	74	76						3
Israel/Western Galilee/ - (75, 73)	EU678763	75	73							2
Israel/Western Galilee/ - (77, 3, 38)	Molad et al, 2009	77	3	38						3
Israel/Western Galilee/ - (77, 4, 3)	EU678762	77	4	3						3
Israel/Western Galilee/ - (78, 25^2, 15^2)	EU678764	78	25	25	15	15				5
Italy/Sicily/ C - (5, Γ^3)	AY702928	5	Γ	Γ	Γ					4
Italy/Sicily/ C - (5, φ^3)	AY702929	5	φ	φ	φ					4
Italy/Sicily/ G - (6, 7^3)	AY702932	6	7	7	7					4
Italy/Sicily/ G - (M^3, Q)	AY702926	M	M	M	Q					4
Italy/Sicily/ G - (Q, M, Q^2, M)	AY702930	Q	M	Q	Q	M				5
Italy/Sicily/ G - (Q, N^3)	AY702931	Q	N	N	N					4
Japan/Okinawa/ - (80, 73, 81, 73, M)	FJ226456	80	73	81	73	M				5
Mexico/ Santa Martha/ - (α, β^3, Γ)	EF053268	α	β	β	Γ	β				5
Mexico/ Tamaulipas/ - (69, 61, 70, 71, 61)	EU283852	69	61	70	71	61				5
Mexico/Aguascalientes/ E - (4, 9, 10, 11, 9)	DQ501243	4	9	10	11	9				5
Mexico/Morelos/ E - (α, β^2, Γ)	AF345869	α	β	β	Γ					4
Mexico/Pichucalco/ E - (α, β, β, Γ^2)	DQ501244	α	β	β	Γ	β	Γ			6
Mexico/Playa Vicente/ - (T, C, B^2, C, B, C)	JN564636	T	C	B	B	C	B	C		7
Mexico/Puente de Ixtla/ G - (12, 13, 14)	DQ501242	12	13	14						3
Mexico/Tamaulipas/ - (28, 29, 104, 29, M, F)	JN564643	28	29	104	29	M	F			6
Mexico/Tamaulipas/ - (4, 63^2, 27, 12)	EU283850	4	63	63	27	12				5
Mexico/Tamaulipas/ - (4, 9, 10^2, 9)	EU283848	4	9	10	10	9				5

Mexico/Tamaulipas/ - (56, 22, 58, 59)	EU283847	56	22	58	59					4
Mexico/Tamaulipas/ - (60, 61^2, 62, 61)	EU283849	60	61	61	62	61				5
Mexico/Tamaulipas/ - (64, 65, D, 65, E)	EU283853	64	65	D	65	E				5
Mexico/Tamaulipas/ - (67, 68, 63, 27, 12)	EU283851	67	68	63	27	12				5
Mexico/Tamaulipas/ - (D, 65, D, 65, E)	EU283854	D	65	D	65	E				5
Mexico/Tepic/ - (α, β^2, Γ)	JN564639	α	β	β	Γ					4
Mexico/Tlapacoyan/ - (103, C, F)	JN564638	103	C	F						3
Mexico/Tlapacoyan/ - (108, β^3, Γ)	JN564641	108	β	β	β	Γ				5
Mexico/Tlapacoyan/ - (T, C, B^2, C^2)	JN564644	T	C	B	B	C	C			6
Mexico/Yucatan/G - (T, C, B^2,C, B, π)	AF345871	T	C	B	B	C	B	π		7
Nigeria/Zaria/ - (54, 55, F)	EU106083	54	55	F						3
Puerto Rico/Puerto Rico/ C - (E, φ^5)	AY191826	E	φ	φ	φ	φ	φ			6
Republic of the Philippines/Cebu/ E - (46, 88,46)	JQ839006	46	88	46						3
Republic of the Philippines/Cebu/K-(107, M, 107, M^2)	JQ839007	107	M	107	M	M				5
Republic of the Philippines/Cebu/D - (46^2)	JQ839005	46	46							2
Republica of China/Taiwan/ - (106, F^2, M^3,F, M)	FJ188386	106	F	F	M	M	M	F	M	8
Republica of China/Taiwan/ - (M, β, Γ, β^5,)	FJ195757	M	β	Γ	β	β	β	β	β	8
Republica of China/Taiwan/ - (α)	FJ188391	α								1
South Africa/EC22/G – (27, 13, 4^2, 37)	KC470183	27	13	4	4	37				5
South Africa/EC23/G – (151, 152, 4^2, 153)	KC470184	151	152	4	4	153				5
South Africa/GP_C1/ - (82, 13, 4^2, 37)	KC470167	82	13	4	4	37				5
South Africa/GP_C2/ - (34, 27, 3, 38, 13, 3, 38)	KC470168	34	27	3	38	13	3	38		7
South Africa/GP_C5/ - (3, 4^3, 37)	KC470169	3	4	4	4	37				5
South Africa/GP-C1112105/G – (34, 37)	KC470170	34	37							2
South Africa/GP-C1817105/G – (34, 13^2)	KC470172	34	13	13						3
South Africa/GP-C4117/G – (3, 36, 38)	KC470171	3	36	38						3
South Africa/GP-C7117105/ - (34, 13, 37)	KC470173	34	13	37						3
South Africa/KZN_14/ E- (142, 43, 25, 31)	KC470179	142	43	25	31					4
South Africa/KZN_19/ E – (141, 140^2)	KC470180	141	140	140						3

South Africa/KZN_D/ - (42, 43, 25, 157, 31)	KC470177	42	43	25	157	31				5
South Africa/KZN_F/ - (42, 43, 25, 31^{2})	KC470174	42	43	25	31	31				5
South Africa/KZN_Y/ - (143, 144, 145, 146)	KC470178	143	144	145	146					4
South Africa/KZN51/E - (147)	KC470182	147								1
South Africa/LP_10/G – (27, 13, 3)	KC470156	27	13	3						3
South Africa/LP34/G – (34, 13, 3, 38)	KC470159	34	13	3	38					4
South Africa/LP37/G – (27, 13^{2}, 37)	KC470153	27	13	13	37					4
South Africa/LP46/G – (3, 38)	KC470154	3	38							2
South Africa/MP_C2/ - (34, 13, 158, 37)	KC470160	34	13	158	37					4
South Africa/MP_C5/ - (15^{2},100, 83)	KC470161	15	15	100	83					4
South Africa/Northeastern Free State/ - (27, 13, 4, 44)	DQ813555	27	13	4	44					4
South Africa/Northeastern Free State/ - (27, 18)	DQ813559	27	18							2
South Africa/Northeastern Free State/ - (27, 37)	DQ813557	27	37							2
South Africa/Northeastern Free State/ - (27, 4, 13, 4^{2})	DQ813554	27	4	13	4	4				5
South Africa/Northeastern Free State/ - (3, 36, 3, 36^{2}, 3, 36, 38)	DQ813564	3	36	3	36	36	3	36	38	8
South Africa/Northeastern Free State/ - (3^{2}, 38)	DQ813547	3	3	38						3
South Africa/Northeastern Free State/ - (33, 35^{2})	DQ813545	33	35	35						3
South Africa/Northeastern Free State/ - (34, 13, 4, 37)	DQ813536	34	13	4	37					4
South Africa/Northeastern Free State/ - (34, 4, 37)	DQ813542	34	4	37						3
South Africa/Northeastern Free State/ - (34, F, 4, H)	DQ813544	34	F	4	H					4
South Africa/Northeastern Free State/ - (39, 37, 13^{4}, 37)	DQ813560	39	37	13	13	13	13	37		7
South Africa/Northeastern Free State/ - (40, Q^{2})	DQ813561	40	Q	Q						3
South Africa/NW_C2/ - (27, 13, 4^{2}, 37)	KC470162	27	13	4	4	37				5
South Africa/NW_C4/ - (27, 13, 4, 37)	KC470163	27	13	4	37					4
South Africa/NW_C5/ - (82, 13, 79, 4, 37)	KC470164	82	13	79	4	37				5
South Africa/NW-C1/G – (34, 13, 3, 36, 38)	KC470165	34	13	3	36	38				5
South Africa/NW-C4/G – (34, 36, 38, 3)	KC470166	34	36	38	3					4
South Africa/Southwestern Free State/ - (27, 13, 4, 13, 4)	DQ813553	27	13	4	13	4				5
South Africa/Southwestern Free State/ - (27, 4, 13^{2}, 37)	DQ813551	27	4	13	13	37				5

South Africa/Southwestern Free State/ - (27, 4^3, 37)	DQ813552	27	4	4	4	37				5
South Africa/Southwestern Free State/ - (3, 13, 4^2, 37)	DQ813550	3	13	4	4	37				5
South Africa/Southwestern Free State/ - (34, 13^2, 37)	DQ813543	34	13	13	37					4
South Africa/Southwestern Free State/ - (34, 45^2, 46, 37)	DQ813556	34	45	45	46	37				5
South Africa/Southwestern Free State/ - (41, 13^3, 4, 37)	DQ813562	41	13	13	13	4	37			6
South Africa/Southwestern Free State/ - (42, 43, 25, 31)	DQ813563	42	43	25	31					4
South Africa/WC10/G – (154)	KC470190	154								1
South Africa/WC11/G – (40, Q^5, 37)	KC470191	40	Q	Q	Q	Q	Q	37		7
South Africa/WC12/G – (27, 13, 37)	KC470192	27	13	37						3
South Africa/WC13/G – (M, Q, M, Q, M)	KC470193	M	Q	M	Q	M				6
South Africa/WC14/G – (155, 36, 38)	KC470194	155	36	38						3
South Africa/WC16/G – (34, 13, 4, 13^2, 4, 37)	KC470196	34	13	4	13	13	4	37		7
South Africa/WC4/ - (40, Q^2, m)	KC470186	40	Q	Q	m					4
South Africa/WC6/G – (3, 4^2, 37)	KC470187	3	4	4	37					4
South Africa/WC7/G - (M^4)	KC470188	M	M	M	M					4
Spain/Va48/ - (40, 47^2, 32, C^2)	DQ811775	40	47	47	32	C	C			6
Taiwan/Nantou/ E - (α, $β^2$, Γ, 105)	EU677384	α	β	β	Γ	105				5
Taiwan/Taichung/ J - (α, $β^3$, Γ, 105)	FJ188389	α	β	β	β	Γ	105			6
USA/California/ G - (B^2, C)	AY010242	B	B	C						3
USA/Cushing/ G - (L, C, B, C)	AY127056	L	C	B	C					4
USA/Cushing2/ G - (K, N^2, F, H)	AY127057	K	N	N	F	H				5
USA/Florida/ G - (A, B^7)	M32871	A	B	B	B	B	B	B	B	8
USA/Glencoe1/ G - (K, F, N, F, H)	AY127053	K	F	N	F	H				5
USA/Glencoe2/ G - (B, M, F, H)	AY127054	B	M	F	H					4
USA/Glencoe3/ G - (T, B, C)	AY127055	T	B	C						3
USA/Idaho/ C - (D^5, E)	M32868	D	D	D	D	D	E			6
USA/Illinois/ G - (M, N, B, M, H)	AF345867	M	N	B	M	H				5
USA/Kansas/ - (B^2)	Palmer et al, 2004	B	B							2
USA/Kansas/ - (B^3)	Palmer et al. 2004	B	B	B						3

USA/Kansas/ - (B^5)	Palmer et al. 2004	B	B	B	B	B						5
USA/Kansas/ - (B^6)	Palmer et al. 2004	B	B	B	B	B	B					6
USA/Kansas/ - (D^2, E)	Palmer et al. 2004	D	D	E								3
USA/Kansas/ - (D^5)	Palmer et al. 2004	D	D	D	D	D						5
USA/Kansas/ - (D^6, E)	Palmer et al. 2004	D	D	D	D	D	D	E				7
USA/Kansas/ - (D^9, E)	Palmer et al. 2004	D	D	D	D	D	D	D	D	D	E	10
USA/Kansas/ - (E, M, φ)	Palmer et al. 2004	E	M	φ								3
USA/Missisippi/ D - (D^4, E)	AY010243	D	D	D	D	E						5
USA/Missouri/ G - (B^4)	AY127052	B	B	B	B							4
USA/NewCastle/ G - (L, B, C, B)	AY127063	L	B	C	B							4
USA/Okeechobee/ G - (L, B, C, B, C)	AY010244	L	B	C	B	C						5
USA/Oklahoma/ G - (U)	AY127059	U										1
USA/Okmulgee/ G - (K, B, V, C)	AY127060	K	B	V	C							4
USA/Oregon/ - (G)	Palmer et al, 2001	G										1
USA/Pawhuska/ G - (I, H)	AY127064	I	H									2
USA/Pawhuska/ G - (K, B, M, F, W)	AY253144	K	B	M	F	W						5
USA/Rassmusen/ - (A, F, H)	AF293064	A	F	H								3
USA/St.Maries/ G - (J, B^2)	AY010245	J	B	B								3
USA/Stigler/ G - (T, B^2, C)	AY127058	T	B	B	C							4
USA/Stillwater/ G - (K, F^3, H)	AY127061	K	F	F	F	H						5
USA/Stillwater/ G - (L, B, C^2)	AY127062	L	B	C	C							4
USA/Stillwater68/ - (K, B, M, F, H)	DQ811776	K	B	M	F	H						5
USA/Texas/ G - (O, B, M, P)	AF428091	O	B	M	P							4
USA/Texas198/ - (B^2, m, B, m)	DQ811778	B	B	m	B	m						5
USA/Virginia/ G - (A, B)	AY010246	A	B									2
USA/Washington/ G - (B^3, C)	M32869	B	B	B	C							4
USA/Wetumka/ G - (K, C, H)	AY010247	K	C	H								3
Venezuela/Barinas/ - (101, B^2, 102)	JF957801	101	B	B	102							4
Venezuela/Barinas/ - (α, $β^4$)	JF957813	α	β	β	β	β						5

Venezuela/Lara/ - (117, 118^{2}, 119, 120)	JF957796	**117**	**118**	**118**	**119**	**120**				5
Venezuela/Lara/ - (121, 122, 123, 124, 125)	JF957787	**121**	**122**	**123**	**124**	**125**				5
Venezuela/Lara/ - (126, 127, 128)	JF957798	**126**	**127**	**128**						3
Venezuela/Lara/ - (129, 130, 131, φ, 57)	JF957792	**129**	**130**	**131**	**φ**	**57**				5
Venezuela/Lara/ - (13, 111, 112, 113)	JF957797	**13**	**111**	**112**	**113**					4
Venezuela/Lara/ - (13, 18^{2}, 13)	JF957805	**13**	**18**	**18**	**13**					4
Venezuela/Lara/ - (136, 137, 138, 139, 95^{2})	JF957810	**136**	**137**	**138**	**139**	**95**	**95**			6
Venezuela/Lara/ - (60, 62, 61, 62, 61, 62, 61)	JF957807	**60**	**62**	**61**	**62**	**61**	**62**	**61**		7
Venezuela/Lara/ - (60, 62^{2}, 61)	JF957808	**60**	**62**	**62**	**61**					4
Venezuela/Lara/ - (64, 96, 97, 99)	JF957809	**64**	**96**	**97**	**99**					4
Venezuela/Lara/ - (64, 98, F^{3})	JF957806	**64**	**98**	**F**	**F**	**F**				5
Venezuela/Lara/ - (84, 62^{2}, 61)	JF957793	**84**	**62**	**62**	**61**					4
Venezuela/Lara/ - (85, 86, 61, 66)	JF957790	**85**	**86**	**61**	**66**					4
Venezuela/Lara/ - (E, φ^{3}, β, 100)	JF957803	**E**	**φ**	**φ**	**φ**	**β**	**100**			6
Venezuela/Lara/ - (T, B^{3})	JF957788	**T**	**B**	**B**	**B**					4
Venezuela/Lara/ - (T, M)	JF957800	**T**	**M**							2
Venezuela/Lara/ - (T, β^{3}, 116)	JF957789	**T**	**β**	**β**	**β**	**116**				5
Venezuela/Lara/ - (α, β, 114, 115, Γ)	JF957791	**α**	**β**	**114**	**115**	**Γ**				5
Venezuela/Merida/ - (13, 63^{2}, 27, 63^{3},27)	JF957802	**13**	**63**	**63**	**27**	**63**	**63**	**63**	**27**	8
Venezuela/Merida/ - (T, B, 90, C, 91)	JF957804	**T**	**B**	**90**	**C**	**91**				5
Venezuela/Merida/ - (T, B, C, F, 89)	JF957814	**T**	**B**	**C**	**F**	**89**				5
Venezuela/Tachira/ - (37, 87, 92,15)	JF957794	**37**	**87**	**92**	**15**					4
Venezuela/Yaracuy/ - (132, 133, 134, 135^{2})	JF957812	**132**	**133**	**134**	**135**	**135**				5
Venezuela/Yaracuy/ - (64, 65, D, E)	JF957811	**64**	**65**	**D**	**E**					4
Venezuela/Yaracuy/ - (93, 10, 94, 109, 110)	JF957799	**93**	**10**	**94**	**109**	**110**				5
